# The Rapid Implementation Feedback (RIF) report: real-time synthesis of qualitative data for proactive implementation planning and tailoring

**DOI:** 10.1186/s43058-024-00605-9

**Published:** 2024-06-21

**Authors:** Erin P. Finley, Joya G. Chrystal, Alicia R. Gable, Erica H. Fletcher, Agatha Palma, Ismelda Canelo, Rebecca S. Oberman, La Shawnta S. Jackson, Rachel Lesser, Tannaz Moin, Bevanne Bean-Mayberry, Melissa M. Farmer, Alison Hamilton

**Affiliations:** 1 Center for the Study of Healthcare Innovation, Implementation, and Policy (CSHIIP), VA Greater Los Angeles Healthcare System, Los Angeles, CA USA; 2 Joe R. & Teresa Lozano Long School of Medicine, The University of Texas Health Science Center at San Antonio, San Antonio, TX USA; 3 David Geffen School of Medicine, University of California Los Angeles, Los Angeles, CA USA

**Keywords:** Rapid qualitative methods, Tailoring, Implementation strategies, Implementation planning, Evidence-based practice

## Abstract

**Background:**

Qualitative methods are a critical tool for enhancing implementation planning and tailoring, yet rapid turn-around of qualitative insights can be challenging in large implementation trials. The Department of Veterans Affairs-funded EMPOWER 2.0 Quality Enhancement Research Initiative (QUERI) is conducting a hybrid type 3 effectiveness-implementation trial comparing the impact of Replicating Effective Programs (REP) and Evidence-Based Quality Improvement (EBQI) as strategies for implementing three evidence-based practices (EBPs) for women Veterans. We describe the development of the Rapid Implementation Feedback (RIF) report, a pragmatic, team-based approach for the rapid synthesis of qualitative data to aid implementation planning and tailoring, as well as findings from a process evaluation of adopting the RIF report within the EMPOWER 2.0 QUERI.

**Methods:**

Trained qualitative staff conducted 125 semi-structured pre-implementation interviews with frontline staff, providers, and leadership across 16 VA sites between October 2021 and October 2022. High-priority topic domains informed by the updated Consolidated Framework for Implementation Research were selected in dialogue between EMPOWER 2.0 implementation and evaluation teams, and relevant key points were summarized for each interview to produce a structured RIF report, with emergent findings about each site highlighted in weekly written and verbal communications. Process evaluation was conducted to assess EMPOWER 2.0 team experiences with the RIF report across pre-implementation data collection and synthesis and implementation planning and tailoring.

**Results:**

Weekly RIF updates supported continuous EMPOWER 2.0 team communication around key findings, particularly questions and concerns raised by participating sites related to the three EBPs. Introducing the RIF report into team processes enhanced: team communication; quality and rigor of qualitative data; sensemaking around emergent challenges; understanding of site readiness; and tailoring of REP and EBQI implementation strategies. RIF report findings have facilitated rapid tailoring of implementation planning and rollout, supporting increased responsiveness to sites’ needs and concerns.

**Conclusions:**

The RIF report provides a structured strategy for distillation of time-sensitive findings, continuous team communication amid a complex multi-site implementation effort, and effective tailoring of implementation rollout in real-time. Use of the RIF report may also support trust-building by enhancing responsiveness to sites during pre- and early implementation.

**Trial registration:**

Enhancing Mental and Physical Health of Women Veterans (NCT05050266); https://clinicaltrials.gov/study/NCT05050266?term=EMPOWER%202.0&rank=1

Date of registration: 09/09/2021.

**Supplementary Information:**

The online version contains supplementary material available at 10.1186/s43058-024-00605-9.

Contributions to the literature

- Tailoring implementation strategies for specific site needs is often critical for successful implementation. However, few approaches ensure that implementation teams possess the necessary information to deliver timely, tailored strategies in multi-site trials.
- We introduce a practical approach, the Rapid Implementation Feedback (RIF) report, designed to share critical information within implementation and evaluation teams. We illustrate how the RIF report has proven instrumental in fostering effective communication and tailoring within the EMPOWER 2.0 Quality Enhancement Research Initiative (QUERI).
- The RIF report offers a method for sharing pertinent and time-sensitive findings, empowering teams to swiftly and effectively tailor implementation in real time.

## Background

As implementation science has matured, implementation trials have become increasingly complex, often comparing two or more implementation strategies, integrating multiple quantitative and qualitative methods, and occurring across a dozen or more sites. Such complex initiatives require larger teams of implementation researchers and practitioners to conduct, raising challenges for effective and timely communication within teams. Meanwhile, tailoring interventions and implementation rollout to align with the unique strengths and challenges at individual sites – recognized as a valuable and often requisite strategy for achieving implementation and sustainment [1–3] – requires intensive, flexible, and dynamic engagement with sites. Contextual factors must be assessed, key partners identified, and critical information synthesized and shared to allow for rapid sensemaking and problem-solving.

The growth of implementation science as a field has been accompanied by an acceleration in the variety, rigor, and rapidity of qualitative methods available to support real-world research translation [4, 5]. Effective work in implementation often requires gathering information that is purposeful and systematic, represents a variety of partners and perspectives, and accurately synthesizes diverse viewpoints to support meaningful communication and decision-making at every stage of implementation. Accordingly, an array of methodological strategies for supporting participatory and partner-engaged processes [6, 7], rapid qualitative data collection and analysis [8, 9], and ethnographic and observational approaches [10–12] have emerged, offering a growing array of qualitative methods to meet the needs of a given study or initiative.

To make use of these methods effectively, work and team processes suitable for the implementation context are needed. The importance of strong communication and relationship networks within implementing sites and teams has been recognized since the early days of the field [13–15], and recent scholarship has examined how relational communication is embedded within most strategies for implementation [16], trust-building [17], and scale-up and spread [18]. Yet relatively little scholarship has put forward methods for ensuring timely and effective communication within implementation teams, particularly amid efforts to achieve site-level tailoring in real-time. Across eight years of conducting hybrid effectiveness-implementation trials in support of improved care delivery for women Veterans, our team has learned that effective tailoring requires capturing and sharing critical information in an ongoing way [4, 10, 19]. In the first part of this article, we describe the development of a pragmatic, team-based approach for the rapid synthesis of qualitative data to support implementation planning and tailoring: the Rapid Implementation Feedback (RIF) report. In the latter part, we describe findings from a process evaluation of adopting the RIF report within the EMPOWER 2.0 QUERI, outlining how use of this approach has evolved our work.

## Methods

### Background and study overview

Women Veterans represent the fastest-growing proportion of VA healthcare users. Despite substantial VA investment in women’s health, gender disparities persist in certain health outcomes, including cardiovascular and metabolic risk and mental health [20–22]. In tailoring healthcare delivery for women, prior studies suggest that women Veterans prefer gender-specific care and telehealth options [19, 23]. In response, the VA EMPOWER 2.0 QUERI is conducting a hybrid type 3 effectiveness-implementation trial [24] comparing the impact of Replicating Effective Programs (REP) and Evidence-Based Quality Improvement (EBQI) as strategies for implementing three virtual evidence-based practices (EBPs) for women Veterans in 20 VA sites across the United States: (1) Diabetes Prevention Program (DPP) to reduce risk of progressing to type 2 diabetes [25]; (2) Telephone Lifestyle Coaching (TLC) to reduce cardiovascular risk [26]; and (3) Reach Out, Stay Strong Essentials (ROSE) to prevent postpartum depression [27]. REP combines phased intervention packaging, tailoring, training and technical assistance, and re-customization during maintenance/sustainment [28], while EBQI offers a systematic quality improvement method for engaging frontline providers in improvement efforts via tailoring, multi-level partnership, and ongoing facilitation [29]. We selected these bundled implementation strategies, REP and EBQI, based on their strong evidence for effectively supporting implementation in diverse healthcare settings [28, 30]. Both of these strategies draw upon pre-implementation needs assessment and planned tailoring as key activities for successful implementation, which we postulated would be important based on our experience in the prior EMPOWER QUERI (2015–2020) [19, 30]. These activities were deemed to be non-research by the VA Office of Patient Care Services prior to funding.

To coordinate the separate implementation and evaluation elements of our work, we established distinct-but-overlapping teams under the broader umbrella of EMPOWER 2.0, dedicated to: (1) implementing each of the EBPs (DPP, TLC, ROSE), with these smaller teams led by principal investigators for each EBP; (2) providing REP- or EBQI-consistent implementation support at each site (i.e., “REP team” and “EBQI team” project directors); and (3) executing qualitative and quantitive components of our overall evaluation (described in detail in [24]), in the form of the “qualitative team” and “measures team,” respectively.

### EMPOWER 2.0 engagement and outreach

**Fig. 1:**
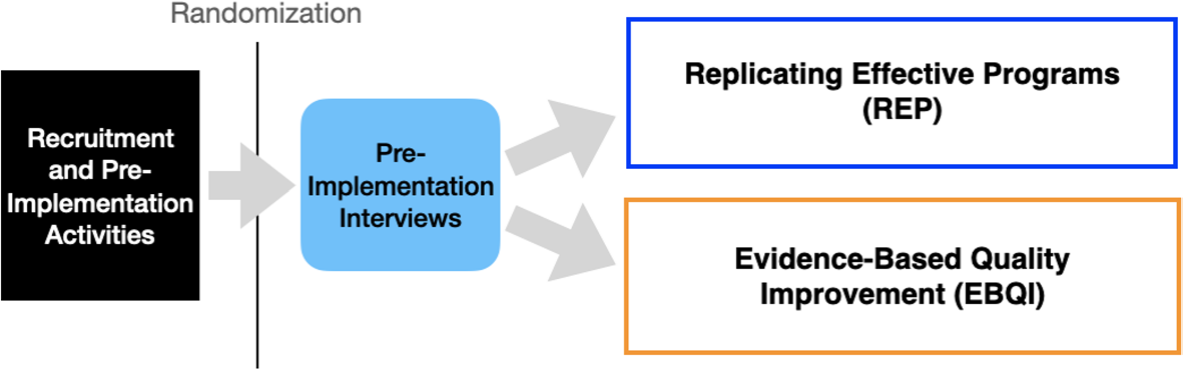
EMPOWER 2.0 QUERI site-level outreach, randomization, and engagement

Working in concert across these implementation and evaluation teams, EMPOWER 2.0 followed a standardized process for engaging with sites (Fig. 1). Initial efforts (beginning pre-funding) involved reaching out to partners at the regional Veterans Integrated Service Network (VISN) level to introduce the EBPs, answer questions, and request a list of potential VA medical centers (VAMCs) within the VISN that might be appropriate for implementation. Following EMPOWER 2.0’s cluster-randomized study design, VISNs were assigned to participate in two of the EBPS (either TLC and ROSE or DPP and ROSE; ROSE was offered to all sites in an effort to ensure an adequate number of pregnant Veteran participants) [24]. We extended invitations to identified VAMCs to participate in the two EBPs available in their VISN. If sites expressed interest, we conducted an introductory meeting with providers and leadership from Primary Care, Women’s Health, Mental Health, Whole Health [31], and/or Health Promotion and Disease Prevention, as appropriate to the EBP and each site’s local organization of care. Once a site confirmed their participation, they were randomized to receive either the REP or the EBQI implementation strategy. Following randomization, they were asked to identify a point person for each EBP and key individuals who would be likely to participate in local EBP implementation teams and/or play an important role in supporting implementation (e.g., VAMC leadership). These individuals (e.g., Medical Director, Health Educator) were then invited to participate in pre-implementation interviews prior to initiating REP or EBQI at their site. In each VISN, partners at the VISN level were also invited to participate in pre-implementation interviews, to obtain broader perspectives on the regional women’s health context and priorities.

### Pre-implementation qualitative interviews

Intended to assess sites’ needs and resources and enable pre-implementation tailoring prior to launch, EMPOWER 2.0 pre-implementation interviews examined baseline care practices for each relevant care condition (prediabetes for DPP; cardiovascular risk for TLC; perinatal mental health for ROSE), as well as updated Consolidated Framework for Implementation Research (CFIR) domains including inner and outer setting, innovation, individuals (e.g., characteristics: motivation) and implementation process [32]. Semi-structured interview guides (previously published [24]) were developed building on prior work in the original EMPOWER QUERI [30] and the Women’s Health Patient-Aligned Clinical Team trial [33]. We have an expert qualitative team, each of whom has master’s or PhD-level training in qualitative methods and years of experience in conducting team-based qualitative research, including using rapid qualitative analysis approaches [8, 9]. Most team members have worked together on EMPOWER and other projects for over five years.

Between October 2021 – October 2022, the qualitative team completed 125 interviews across 16 sites, with site and VISN-level participants representing a range of roles, including Women Veteran Program Managers, Women’s Health Primary Care Providers, Maternity Care Coordinators, primary care team members, health coaches, and nutritionists. Pre-implementation interviews took an average of 57 days (range 15–108 days) to complete per site, and included 4–13 participants depending on the size and complexity of the care facility.

### Developing the Rapid Implementation Feedback (RIF) report

The EMPOWER 2.0 qualitative team has a well-established approach to conducting rapid qualitative analysis [8, 19] and strong personnel infrastructure and expertise. Even so, once pre-implementation interviews began, challenges quickly arose in ensuring that findings were being communicated to EMPOWER 2.0 implementation teams for DPP, TLC, and ROSE in a timely and effective manner, particularly given that each team was working with multiple sites concurrently. Key questions included: how do we ensure early findings are shared in time to support pre-implementation tailoring? How do we communicate effectively across the qualitative team conducting interviews and the teams responsible for implementation? And how do we keep qualitative team members up-to-date on implementation, so they are well-informed for interviews?

In responding to these challenges, we developed the Rapid Implementation Feedback (RIF) report to support data distillation and bidirectional feedback across our qualitative and implementation teams. In developing the RIF, the EMPOWER 2.0 implementation teams, which are composed of investigators and project directors for each EBP who provide external implementation support for each site, met with the qualitative interview team and agreed upon high-priority topic domains to be extracted from the interviews. These domains were related to implementation planning and included *critical roles for implementation planning and launch*; *implementation concerns and/or demand for the EBP*; and *use of data to track women Veterans’ population health needs* (see Table 1). These topics reflected both specific CFIR subdomains included in the pre-implementation interview guide (e.g., *use of data* as an assessment of the CFIR subdomain for *information technology infrastructure*), as well as higher-level domains combined to aid in prioritizing key issues (e.g., germane responses related to *inner setting*, *individual characteristics*, and *implementation process* were combined into *implementation concerns*). These topic domains were used to create a RIF report template (see Appendix 1), which was organized under headings by *VISN* (outer setting), *site* (inner setting), and *EBP* [32]; the same domains were selected for all EBPs, ensuring consistency in data distillation across the project. Compiling the RIF report ensured that, for example, all interview data relevant to critical roles for implementation planning for ROSE in Site A were collated and easy to locate. Thereafter, at the conclusion of an interview, the qualitative team reviewed interview notes and/or Microsoft Teams transcripts and extracted key points relevant to each priority topic; in doing so, team members followed a process similar to that used in developing structured summaries for rapid qualitative analysis [8, 34], but differing by a targeted focus on relatively few domains. For each interview, the analyst would summarize key points related to each RIF domain (e.g., *critical roles for implementation planning and launch*), as well as any brief or particularly salient quotes; every key point or quote was also labeled with a study identification number indicating the role of the respondent. The resulting key points and quotes were then added to the RIF report, creating a single, up-to-date written resource for implementation teams, which was cumulatively updated over time.

**Table 1:** Sample EMPOWER 2.0 interview questions and corresponding Rapid Implementation Feedback (RIF) report domains

Sample EMPOWER 2.0 Interview Questions	Corresponding Rapid Implementation Feedback (RIF) Report Domains	Sample Site-Level Findings
**General site-level information**		
We’re interested in how different sites use data to monitor patient health status. Are there specific tools, dashboards, or data sets that you use to monitor Veteran patient health status? Who uses these data and how do they use it?	Tools, dashboards, and data sets used	• Women’s Health Sharepoint, used by the Women’s Health Medical Director • Primary Care Dashboard, used by Women’s Health teams to track appointments & performance metrics
Are you aware of specific performance metrics related to Women’s Health? If so, what are they (are they local, regional, national) and do you or someone else monitor them?	Performance metrics related to Women’s Health	• Maternal Care & Fetal Outcomes • Mammography • Cervical Care • Diabetes • Hypertension
**Evidence-Based Practice (EBP)-specific information** ***Sample EBP:* Reach Out, Stay Strong Essentials (ROSE)**		
What service lines and roles do you think are critical for standing up ROSE at this site/VISN?	Critical service lines for implementation	• Mental Health • Women’s Health • Primary Care • Social Work • Health Promotion Disease Prevention • Whole Health
	Critical roles for implementation	• Women’s Mental Health Champion • Maternity Care Coordinator • Women’s Health Medical Director • Women Veteran Program Manager
How do you feel about the idea of adding ROSE at your site/VISN? Do you feel ROSE implementation will work well at your site/VISN? Why or why not? What concerns do you have about ROSE in your setting?	Implementation concerns	• Staffing: Facility is understaffed; staff are implementing other programs • Intervention length: 90 min group sessions seem long, especially for new moms, suggest modifying to 60 minutes • Logistics: Identify who will lead sessions; clarify if sessions recurrent or on rolling basis; challenges scheduling virtual sessions • Women Veterans: Recruitment & engagement has been a concern for other groups; childcare could be barrier to attendance
Do you think there is demand for a program like ROSE at your site/VISN?	Implementation demand	• Staff motivation: Many dedicated staff have embraced the idea of ROSE; staff could be incentivized to do the training and run groups • Patient demand: Women Veterans have requested more postpartum support, including more support groups • Aligns with VA priorities: Maternity care is a national priority

This approach to analysis is distinct in two key ways from the data distillation process typically used in rapid qualitative analysis [8, 34–36]. First, in rapid qualitative analysis, templated summaries are first created at the level of the individual interview or other data episode, so that each data episode is associated with a summary of contents that can later be compiled into a multi-episode matrix. Second, structured summaries are traditionally intended to capture *all* of the key findings in a given data episode, and thus are both more comprehensive and less focused than the RIF report. By contrast, the RIF report collapsed two steps (i.e., summary then matrix) into one (i.e., RIF report) to assemble a targeted selection of high-priority data. In addition, because the data for each domain were collated from the beginning into a single document, the process of assessing data heterogeneity (e.g., diversity of opinions) and adequacy (e.g., saturation) for a given site was expedited. Up-to-date findings could be made available to the implementation teams on a consistent basis, despite the fact that the qualitative team was often interviewing among multiple sites concurrently. During this period, EMPOWER 2.0 held a weekly full-team meeting to coordinate implementation and evaluation efforts. The day before this weekly meeting, the updated RIF report was sent to the full EMPOWER 2.0 team in a secure encrypted email, with new additions highlighted for easy reference; the team was also notified if there were no RIF updates for the week. As implementation teams were also working concurrently across multiple sites, the RIF report became a centralized resource for organizing essential information in a dynamic environment.

**Fig. 2:**
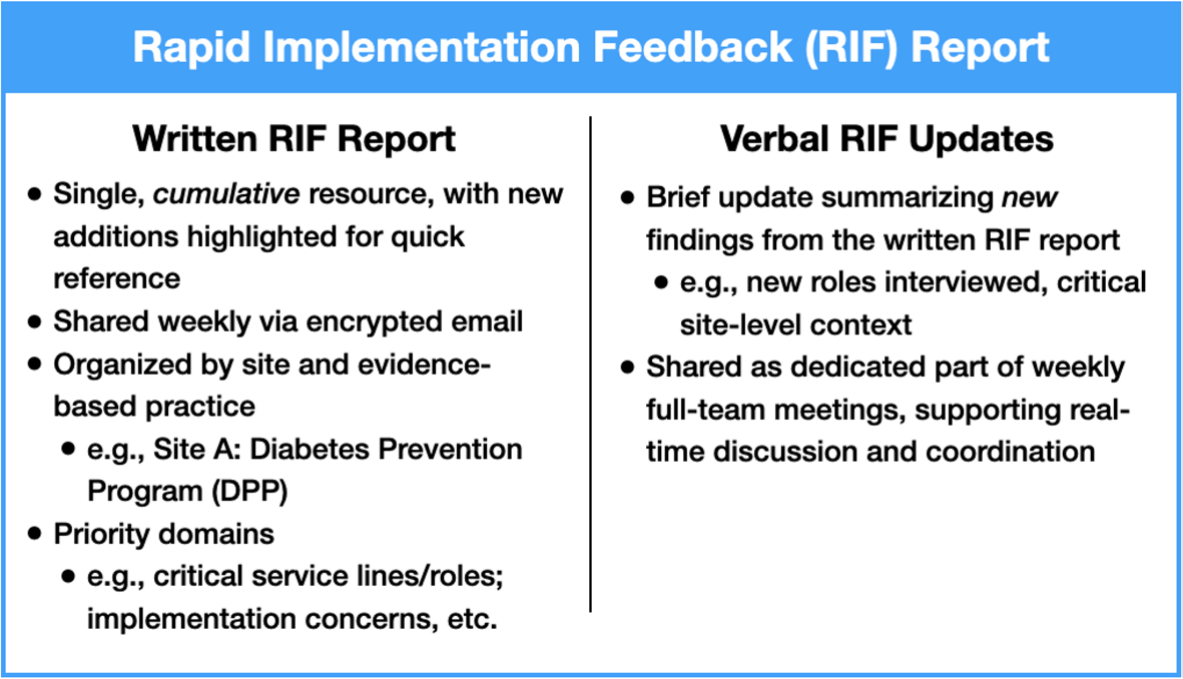
Core components of the Rapid Implementation Feedback (RIF) report

Although the brief written RIF expedited communication of time-sensitive information across teams, challenges continued to arise in coordinating activities, tailoring EBPs, and general communication with sites. We therefore added a verbal update to the RIF Report (see Fig. 2), summarizing new additions to the RIF as part of our overall EMPOWER 2.0 weekly meeting. Updates were brief, organized by site, and included a brief summary of interviews conducted that week, along with the roles interviewed and unique findings (e.g., staff turnover issues). Members of the qualitative team also gave feedback on whether saturation had been reached at a site, or if additional interviewing would be helpful in developing a snapshot of key site features, strengths, and potential challenges.

### Process evaluation

To assess whether the RIF was an effective method for communication and coordination, we conducted a process evaluation of EMPOWER 2.0 teams’ experiences of using the RIF report. We reviewed periodic reflections conducted by the first author as part of EMPOWER 2.0’s overall implementation evaluation with 11 members of five internal teams: those responsible for leading DPP, TLC, and ROSE implementation (i.e., PIs and Co-PIs), and for supporting sites using REP and EBQI implementation strategies (i.e., project directors). Periodic reflections [10] are lightly guided discussions conducted by phone or teleconference software, which allow for consistent documentation of implementation activities, processes, and events, both planned and unexpected. We adapted the original periodic reflection template [10] as a discussion guide for EMPOWER 2.0 (previously published [24]). Reflections lasted 15–60 minutes, with length roughly corresponding to the amount of recent implementation activity, and were conducted monthly or bi-monthly with each team.

In examining how the RIF report was working for our teams, we conducted thematic analysis [37] of all periodic reflections (*n* = 32) completed with EMPOWER 2.0 teams between October 2021, when the RIF was first introduced, and October 2022. All text relevant to the RIF report was extracted and reviewed inductively for key themes associated with perceived impacts of the RIF, resulting in a preliminary set of emergent themes, which were codified into a codebook. All segments of extracted text were then reviewed again and assigned codes as appropriate to their meaning; central findings for each code/theme were then distilled. This preliminary analysis was conducted by the lead author and then presented back to the full EMPOWER 2.0 team to allow for debriefing and member checking [38, 39] over a series of meetings. Team members provided substantive feedback that aided in refining themes, and offered additional reflection and commentary on the RIF report and its role within team processes.

## Results

We identified five interconnected impacts associated with introducing the RIF report into EMPOWER 2.0 team processes: enhanced communication across teams; improved quality and rigor of qualitative data; heightened sensemaking around emergent challenges; increased understanding of site readiness; and informed tailoring of REP and EBQI implementation strategies. We describe each of these in turn below.

### Enhanced communication across teams

As intended, the RIF was felt to be an effective strategy for improving communication across EMPOWER 2.0’s internal teams. Having the RIF available in written format created an easily accessible resource for implementation teams as they prepared for next steps in engaging with sites, and for qualitative team members as they prepared for upcoming interviews. The verbal RIF update, because it occurred alongside implementation team updates as part of the weekly team call, ensured that information-sharing was bidirectional in real time. The continuous flow of information provided a regular opportunity for answering questions, clarifying areas of potential confusion, and identifying where additional information was needed. Additionally, the RIF served to keep all team members in sync with site-specific information on an ongoing basis.

“I love that the qualitative team is giving us real-time feedback. I don’t think I’ve ever done that except informally. I think that’s been a really nice addition to our meetings.” [EBP 1 lead]

On the whole, the enhanced communication among teams was felt to support team “synergy” and increase synchronization of activities in continued data-gathering and site engagement.

### Improved quality and rigor of qualitative data

Although improving rigor was not an explicit goal of developing the RIF report, introducing this structured process was felt to have improved both the quality of data collection and the rigor of early analyses. Because of the improved bidirectional communication occurring as part of the weekly verbal RIF report with implementation teams, qualitative team members felt as though they had an increased understanding of implementation activities and site-level context. This in turn was felt to improve the quality of their interviewing by allowing them to ask more attuned follow-up questions and to prioritize topics that were “meaningful to inform implementation.”

“[We] felt very disconnected in the beginning like we didn’t have any information. Having the weekly calls to talk about these things was really helpful.” [Qualitative team member 1]

Qualitative team members also reported feeling more consistent and “in sync” in their processes for interviewing and preparing the RIF report, as the weekly discussions provided an opportunity for the team to observe, confer, and calibrate regarding the conduct of interviews and the content and level of detail included in ongoing RIF updates.

“It helps us stay impartial as interviewers across stakeholders, across sites, and as we modify the interview guide. It kept all of us…aligned with the parts we need to dig deeper into because they’re RIF/high priority.” [Qualitative team member 2].

In addition, introducing the RIF report was felt to increase the trustworthiness of preliminary analyses and data distillation, because while initial data reviews can be impressionistic or anecdotal, the RIF provided a structured and systematic way of consolidating multi-site data from the first pass. Because the RIF report provided early synthesis, it also aided in generating ideas for targeted analysis and coding conducted as part of evaluation activities in later phases.

### Heightened sensemaking around emergent challenges

Arising out of the enhanced team communication, and perhaps supported by the improved quality of information being gathered and distilled by the qualitative team, discussions prompted by the RIF helped the EMPOWER 2.0 team to identify and develop solutions to emergent challenges. As one example, the qualitative team quickly realized that, while it is common practice to keep implementation-focused and evaluation-focused teams distinct in an effort to reduce bias in hybrid trials, sites viewed everyone associated with EMPOWER 2.0 – including interviewers – as an “ambassador” of the project. Interviewers found early on that they were fielding important questions from sites regarding the EBPs and/or implementation plans, and often lacked the information to provide an appropriate response, which placed them in an awkward position. After this issue was raised as part of a weekly RIF update, the teams worked together to develop a living *Frequently Asked Questions* document to help interviewers answer common questions that were coming up during interviews. This document was later helpful in standardizing communication with sites more generally, serving as a resource for implementation teams as well.

In a second example, a key pre-implementation effort by the EMPOWER 2.0 measures team involved developing a dashboard of population health and performance metrics tailored to provide actionable information to sites on the healthcare needs of their women Veterans. As preparations for site launch continued, and discussions of RIF findings informed ongoing planning efforts, the measures team realized they lacked information on how sites were using existing population health and performance measures. The measures and qualitative teams then worked together to update the interview guide and add priority domains to the RIF report to aid in dashboard development. Having integrated these additions, the qualitative team was able to rapidly confirm the need for a dashboard display of women-only performance measures, and data were used to support tailoring to sites’ needs.

### Increased understanding of site readiness

Reflecting the enhanced communication and improved data quality associated with adopting the RIF report, the EMPOWER 2.0 teams were also more able to develop timely assessments of site readiness. The distillation of qualitative interview data provided important contextual information about site-level participants’ level of EBP awareness, motivation, and competing demands prior to implementation planning meetings.

“They just seem generally enthusiastic.” [EBP 2 lead]

“Most of what I was picking up on was people saying, ‘We don’t have anyone *to do* it.’ Just sites saying that they don’t have people…they don’t want to take it on right now.” [EBP 3 lead]

Readied with this information, implementation teams were able to prepare for engagement and planning efforts with a greater understanding of what the critical issues were likely to be.

### Informed tailoring of REP & EBQI strategies

Finally, building on an improved understanding of sites’ pre-implementation readiness, EMPOWER 2.0 teams felt better equipped to engage in planned tailoring of site outreach and implementation activities within the REP and EBQI strategy bundles. For example, when a key leader at one site was revealed to be “not entirely on board” with DPP implementation, the DPP team lead was able to offer targeted outreach to acknowledge and address the concerns expressed. When concerns were raised about staffing and EBP ownership prior to launch of ROSE, the ROSE team lead expressed, “We were prepared for tough conversations.”

“That became our ‘MO’…anything that comes up [in the RIF], we’ll try to address in the kick-off [meeting with sites] to show that we’re helping in addressing their questions.” [EBP 1 lead]

## Discussion

The RIF report was developed in response to the challenge, within the EMPOWER 2.0 hybrid type 3 effectiveness-implementation trial, of distilling and sharing critical information among internal teams as they pursued distinct implementation and evaluation tasks with an evolving cast of dynamic sites. Combined, the RIF report’s written and verbal components provide a method and process for rapidly extracting high-priority, actionable data, sharing these data in a focused and digestible way, and supporting team sensemaking and tailoring of implementation approaches in real time.

In evaluating the RIF report process, we found that its key benefits were interconnected and mutually reinforcing. Bidirectional communication increased the quality of qualitative data collection, which in turn improved the depth and salience of the data conveyed to the implementation teams, which in turn increased the teams’ ability to engage in active sensemaking and identify effective strategies for tailoring the implementation approach at each site. The tight informational feedback loop allowed us to be nimble and iterative both in data-gathering (e.g., by adding novel domains to the RIF as needed) and in tailoring (e.g., by allowing us to customize early messaging to address sites’ most pressing concerns).

Tailoring and adaptation of both interventions and implementation strategies have been recognized as essential for the successful translation of research into routine practice [40–43]. In response, a variety of qualitative and mixed-methods approaches have been put forward for capturing feedback from diverse partners, including user-centered adaptation [44], the Method for Program Adaptation through Community Engagement (M-PACE) [45], the ADAPT guidance [46], concept mapping [47], and intervention mapping [48]. These approaches have strengthened capacity for implementation researchers and practitioners to gather and synthesize often wide-ranging perspectives into actionable guidance for improving the acceptability, feasibility, appropriateness, and compatibility of interventions and implementation strategies. Yet there remains significant opportunity to streamline and systematize methods for tailoring in the context of hybrid type 2 and 3 trials, which often conduct formative evaluation in real time amid simultaneous data collection and implementation activities. In addition to providing a model for how to embed a structured method for data capture, distillation, and sharing within a complex implementation trial, we believe the RIF report offers a pragmatic method to improve both the quality of information synthesis and the ability of teams to engage in timely sensemaking.

Creating an effective internal communication process via the RIF supported tailored delivery of EBPs at each site, which in turn was felt to enhance the relationships between EMPOWER 2.0 QUERI members and site partners. The role of relationships as an underlying and underexplored element within implementation has garnered increasing attention [15]. Bartley et al. [16] conducted an analysis of the Expert Recommendations for Implementing Change (ERIC) taxonomy of implementation strategies [49], and found that nearly half (36 of 73) could be classified as highly or semi-relational in nature. Connelly and collaborators [50] developed a Relational Facilitation Guidebook based in relational coordination and the principle that high-quality communication and relationships result in improved healthcare quality. Metz and colleagues [17] have proposed a theoretical model for building trusting relationships to support implementation, drawing on theory and research evidence to identify both technical and relational strategies associated with nurturing trust. There is considerable overlap between Metz et al.’s strategies and the processes supported by adopting the RIF report in EMPOWER 2.0, particularly those related to bidirectional communication, co-learning, and frequent interactions, which in turn enabled greater responsiveness to sites. We found the structured communication offered by the RIF helped to support trust-building both within EMPOWER 2.0 and in our teams’ interactions with sites.

Future teams weighing potential use of the RIF report should first consider whether the RIF report is suitable to their project goals and resources. It may be less suitable for teams whose timelines allow for traditional coding-based or rapid qualitative approaches to data analysis, who do not intend to engage in formative evaluation or planned tailoring, or who have concerns that any modifications to the implementation approach may be incompatible with their trial design. In EMPOWER 2.0, core components for determining fidelity to implementation strategy in both study arms (REP and EBQI) were identified before initiating pre-implementation activities, and both strategies included planned tailoring to address specific conditions at sites (e.g., perceived patient needs, key professional roles and service lines to be involved). We were thus able to ensure that no decisions made in RIF-related or other discussions varied from our trial protocol.

Teams electing to adopt the RIF report may benefit from discussing how best to integrate this method into their workflow, and what specific tailoring of the RIF report is needed to ensure alignment with their implementation, research, and/or evaluation goals. We recommend that teams discuss and come to consensus on four RIF elements: (1) selected high-priority topic domains, e.g., site-level concerns, which may be higher-level or more closely focused on implementation theory constructs, as appropriate to the project; (2) what data sources will be included (e.g., data from provider or leadership interviews, surveys, or periodic reflections); (3) the preferred format for written and verbal RIF reports, including salient categories for organizing information (e.g., by site or professional role); and (4) the preferred frequency of sharing RIF reports. Given the established importance of identifying effective local champions in implementation [51–54], *identifying critical roles and service lines for implementation planning and launch* are domains likely to be of value for many projects, as is the domain of *implementation concerns*, which encapsulates important doubts or anxieties expressed by respondents that may be addressable by the implementation team. Teams documenting shifts to the implementation approach in response to respondent feedback might also consider adding a *tailoring*/*action items* or *next steps* domain to track decisions made during discussions of RIF findings. With regard to frequency, weekly RIF reports worked well for EMPOWER 2.0 because this tempo aligned with existing meetings and the busy pace of pre-implementation activities, but this frequency may not be necessary for all teams. Dialogue across these issues is likely to be of value for teams in developing a shared understanding of how project goals will be operationalized, and may allow for more agile responses when change is needed or challenges arise.

There are several limitations to the process evaluation described here. First, it should be noted that periodic reflections were conducted by the first author, who has worked with most members of the implementation teams for at least five years. As an ethnographic method occurring repeatedly over time, reflections benefit from the long-term relationship built between discussion lead and participants, and may be subject to less reporting bias than other data collection methods [10]. Nonetheless, the potential for biased reporting should be acknowledged. We endeavored to ensure the accuracy, completeness, and trustworthiness of findings [39, 55, 56] by engaging in multiple rounds of member checking with the EMPOWER 2.0 team, first in dedicated meetings and later in preparing and revising this manuscript.

In considering the limitations of the RIF report as a methodological approach to support effective distillation and tailoring, it is important to note that this process was developed and executed by a highly trained and experienced team, which likely facilitated qualitative team members in completing the structured reports in a timely and consistent manner. We found that analyses conducted for the RIF report were adequate to support all of the pre-implementation tailoring required for this initiative; however, projects – and particularly projects occurring earlier in the implementation pipeline than this hybrid type 3 trial – may vary in their early-stage analytic needs. Notably, no negative impacts associated with introducing the RIF were identified by team members; this may reflect the fact that the RIF report replaced other rapid qualitative analysis activities (e.g., developing structured summaries for each interview) rather than adding to the team workload. It should be noted that the EMPOWER 2.0 core team also builds on significant experience working together over time, which may have enhanced the quality of communication and coordination emerging from RIF updates. The RIF report may not be relevant or appropriate in implementation efforts where formative evaluation and/or tailoring are not intended or desirable (e.g., in implementation trials assessing the effectiveness of strategies that do not include planned tailoring), although its step-by-step process for synthesizing data relevant to high-priority topics for rapid communication is likely to have broad utility. Future research should consider whether the RIF report has generalizability as a method for use in less complex implementation studies, or by smaller or less experienced teams.

## Conclusions

Rapid qualitative methods are a critical tool for enhancing implementation planning, communication, and tailoring, but can be challenging to execute in the context of complex implementation trials, such as those occurring across multiple sites and requiring coordination across implementation and evaluation teams. The RIF report extends rapid qualitative methods by providing a structured process to enhance focused data distillation and timely communication across teams, laying the groundwork for an up-to-date assessment of site readiness, improved identification and sensemaking around emergent problems, and effective and responsive tailoring to meet the needs of diverse sites.

### Supplementary Information

Supplementary Material 1.

## Data Availability

The datasets generated and/or analysed during the current study are not publicly available as participants have not provided consent for sharing; de-identified portions may be available from the corresponding author on reasonable request.

## Abbreviations

DPP: Diabetes Prevention Program
EBP: Evidence-Based Practice
EBQI: Evidence-Based Quality Improvement
EMPOWER: Enhancing Mental and Physical Health of Women Veterans through Engagement and Retention
QUERI: Quality Enhancement and Research Initiative
REP: Replicating Effective Programs
RIF: Rapid Implementation Feedback
ROSE: Reach Out, Stay Strong Essentials
TLC: Telephone Lifestyle Coaching
VA: Veterans Affairs
VAMC: VA Medical Center
VISN: Veterans Integrated Service Network
