# Supplementary material for: The Rapid Implementation Feedback (RIF) report: real-time synthesis of qualitative data for proactive implementation planning and tailoring
Source: Implement Sci Commun. 2024 Jun 21;5:69. doi: 10.1186/s43058-024-00605-9 (PMC11191329; doi:10.1186/s43058-024-00605-9)
Supplement: Supplementary file 1 — Supplementary Material 1. [file 43058_2024_605_MOESM1_ESM.docx]

**Appendix 1. EMPOWER 2.0 Rapid Implementation Feedback (RIF) Report Template**

with sample data extracts

**Pre-implementation interviews through [*Date*]**

Site A [*Site*], Region 1 [*Region*], EBQI [*Study Arm*]

*Tools/Dashboards/Data sets used:*

- WVPM does not routinely use Tools/dashboards/datasets [1170111 (*Study Identifier*)]
- Routinely uses BHL (not dashboards) for patient data monitoring [1170110]
- *Etc.*

*Performance metrics related to Women’s Health:*

- Quality management provides some amount of performance metrics attention and relays to WH [1170111]
- *Etc.*

EBP #1: Telephone Lifestyle Coaching (TLC)

*Critical service lines:*

- Whole Health (critical), Primary Care, Behavioral Health [1170108]
- Women’s Health [1170106]
- Women’s Health, MH, Primary Care, Nutrition, Informatics Dept [1170103]
- *Etc.*

*Critical roles:*

- Whole Health Champion, Chief of MH, Outreach Specialists, advisory staff [1170108]
- MH Champions, Outreach Specialists [1170106]
- HPDP Program Coordinator [1170103]
- Health Behavior Coordinator [1170102]
- *Etc.*

*Implementation concerns:*

- Lack of involvement from Women’s Program [1170106]
- TLC might compete with in-house coaching offered by Whole Health [1170101]
  - Not enough staff aware of health coaching; difference unknown between TLC and Whole Health coaching programs
- Will be critical to differentiate between consults to Whole Health vs TLC as part of implementation training to PCPs/PACT [1170104]
- Some concern about possible confusion between consult orders for Whole Health vs TLC [1170102]
- *Etc.*

*Implementation supports/demand:*

- Seen as valuable service that could be offered to Veterans [1170104]
- *Etc.*

EBP #2: Reach Out, Stay Strong Essentials (ROSE)

*Critical service lines:*

- MH & Women’s Health [1170108]
- MH & Women’s Health [1170104]
- Primary Care, MH, Women’s Health [1170113]
- *Etc.*

*Critical roles:*

- Women’s Health Director [1170108]
- Women Veteran Coordinator, Maternity Care Coordinator [1170108]
- Anyone in Women’s Health, and Women’s Mental Health Champions [1170106]
- Pregnancy Outreach Coordinator [1170103]
- Maternity Care Coordinator [1170107]
- *Etc.*

*Implementation concerns:*

- Lack of involvement from Maternity Care Coordinator [1170106]
- Women’s Health staff have declined involvement, including Women’s Health Director [1170108]
- Who/how to refer for ROSE? Will need a new Standard Operating Procedure to fast-track referral from Mental Health to specialty services [1170108]
- Concern over group model for ROSE; historically, women at this site haven’t “bought into groups” [1170109]
- *Etc.*

*Implementation supports/demand:*

- Desire for added programming to support pregnant Veterans [1170109]
- *Etc.*

Site B, Region 1, REP

*Tools/Dashboards/Data sets used:*

- VSSC and VISTA (according to PSA for WH) [1070111]
  - Suggests the MH Coordinator (also the MST Coordinator) uses dashboards routinely
- *Etc.*

*Performance metrics related to Women’s Health:*

- Defers question to MH Coordinator [1070111]
- *Etc.*

*[RIF continues…]*
